# High FCRL5 expression predicts poor treatment response and survival in newly diagnosed multiple myeloma: a retrospective study

**DOI:** 10.3389/fonc.2026.1765006

**Published:** 2026-06-29

**Authors:** Cainan Yu, Minghua Zhang, Jie Hui, Mengqing Zhu, Zengtian Sun, Yueyue Sun, Qian Sun, Zhiling Yan, Feng Zhu, Mingshan Niu, Depeng Li, Kunming Qi

**Affiliations:** Department of Hematology, The Affiliated Hospital of Xuzhou Medical University, Xuzhou, China

**Keywords:** Fc receptor-like 5 (Fcrl5), multiple myeloma, prognosis, survival analysis, treatment outcome

## Abstract

**Objectives:**

Limited research is currently available regarding the prognostic value of Fc receptor-like 5 (FCRL5) in newly diagnosed multiple myeloma (NDMM). This study aimed to investigate the association of FCRL5 expression with treatment response and survival outcomes in NDMM patients, with a goal of providing insights for early risk stratification.

**Methods:**

We retrospectively analyzed a cohort comprising 54 NDMM patients treated between January 2024 and January 2025 at a single center. Pretreatment FCRL5 expression was quantified by flow cytometry, and the median value (75.15%) was utilized to stratify patients into two groups: high expression group and low expression group for comparison of baseline characteristics, treatment response, and survival outcomes.

**Results:**

Baseline characteristics, including age and disease stage, were balanced between the two FCRL5 expression groups, with all *P*-values > 0.05. After four induction cycles, the group with high FCRL5 expression showed substantially lower rates of complete response (25.93% vs. 51.85%), ≥very good partial response (33.33% vs. 70.37%), and minimal residual disease negativity (29.63% vs. 70.37%) (*P* < 0.05 for all). At 10.3-month median follow-up, the median progression-free survival (PFS) (10.6 months in contrast to not reached, *P* = 0.002) and overall survival (OS) (13.2 months as opposed to not reached, *P* = 0.02), as well as 1-year PFS rate (21.43% vs. 76.47%, *P* = 0.004) were markedly shorter. Multivariate regression analysis confirmed high FCRL5 expression as an independent adverse prognostic factor for both PFS (*HR* 7.32, 95% *CI* 1.86 - 28.74, *P* = 0.004) and OS (*HR* 9.82, 95% *CI* 1.01 - 95.10, *P* = 0.049).

**Conclusion:**

High FCRL5 expression was significantly associated with inferior response depth and survival in NDMM. Our findings suggested that FCRL5 may serve as a valuable biomarker for risk stratification, though further prospective validation is required.

## 1 Introduction

Multiple myeloma (MM) is a clonal plasma cell neoplasm that exhibits significant clinical and biological heterogeneity and ranks as the second most common hematologic malignancy worldwide (1, 2). Although therapeutic options have expanded substantially to include next-generation proteasome inhibitors, immunomodulatory agents, bispecific antibodies, and chimeric antigen receptor T-cell immunotherapy (CAR-T), most patients will eventually experience relapse and develop treatment-resistant disease (3, 4). This clinical reality highlights the need for reliable prognostic tools to improve risk stratification at diagnosis and guide personalized treatment strategies. Current predictive performance provides only moderate discriminatory power for newly diagnosed multiple myeloma (NDMM), which incorporates stage of disease and conventional cytogenetic abnormalities. Despite the emergence of biomarkers such as B cell maturation antigen (BCMA) and G protein-coupled receptor class C group 5 member D (GPRC5D), their ability to predict clinical outcomes remains limited (5, 6). Therefore, it is necessary to identify novel biomarkers with independent prognostic value.

Fc receptor-like 5 (FCRL5) is a transmembrane protein restricted to the B lineage and encoded in the 1q21–23 region. It contains both immunoreceptor tyrosine-based activation and inhibitory motifs (ITAMs/ITIMs), enabling it to finely modulate B cell signaling and immune responses (7–9). Unlike conventional B-cell biomarkers such as CD19 and CD20, FCRL5 is minimally detected in normal cells, but is commonly upregulated in myeloma. This highly selective expression pattern, together with its biological function, has positioned FCRL5 as a compelling immunotherapeutic target and a potential prognostic biomarker alongside BCMA and GPRC5D (10–12). Importantly, it should be recognized that the correlation between high FCRL5 expression and poor outcomes may be epiphenomenal. Rather than directly mediating drug resistance, FCRL5 expression might simply capture a broader, more aggressive disease biology or reflect a specific state of clonal evolution in NDMM. Since this study did not include functional or immune landscape analyses, a direct causal link between FCRL5 and treatment failure cannot be established. These results should be viewed as hypothesis-generating, indicating that FCRL5 is a marker of an inherently high-risk disease phenotype.

However, existing evidence regarding the clinical implications of FCRL5 in MM pathogenesis and treatment remains limited. Niu et al.’s research found that elevated FCRL5 expression was linked to more aggressive disease features and inferior survival outcomes for patients with this disease (13). In addition, preclinical investigations have demonstrated the substantial anti-myeloma activity of approaches targeting FCRL5, such as antibody-drug conjugates (ADCs) and CAR-T cells (14). Despite these promising therapeutic developments, some studies questioning FCRL5 as a stand-alone biomarker (15, 16). A critical knowledge gap surrounds FCRL5’s prognostic relevance in NDMM, stemming from the lack of consensus and insufficient real-world clinical evidence. To address this gap, we performed a retrospective analysis of FCRL5 expression and its clinical associations in 54 NDMM patients to assess its utility as a prognostic biomarker for early diagnosis and risk stratification.

## 2 Materials and methods

### 2.1 Study methodology and participants

At the Affiliated Hospital of Xuzhou Medical University, this single-center retrospective study consecutively screened and ultimately enrolled 54 patients with NDMM from January 31, 2024, to January 31, 2025 after the strict application of pre-specified inclusion and exclusion criteria. The inclusion criteria were as follows: (1) confirmed NDMM diagnosis per the 2016 diagnostic criteria established by the International Myeloma Working Group (IMWG) (17); (2) measurable pretreatment FCRL5 expression by flow cytometry; (3) availability of complete baseline clinical data and follow-up data. Exclusion criteria included: solitary plasmacytoma, plasma cell leukemia, other active malignancies, severe psychiatric disorders, insufficient clinical documentation, or those lost to follow-up. This research protocol was approved by the Ethics Committee of the Affiliated Hospital of Xuzhou Medical University (Approval No. XYFY2023-KL216-01). Written informed consent was secured from all participants.

### 2.2 Data collection and definitions

Baseline information included the following: demographics; routine laboratory parameters such as hemoglobin (Hb), albumin (Alb), platelets (PLT), serum β2-microglobulin (β2-MG), serum creatinine (Cr), lactate dehydrogenase (LDH), serum calcium (Ca²^+^); and disease-related features including extramedullary disease (EMD), bone marrow plasma cell proportion (BMPC). Disease staging was determined according to the International Staging System (ISS) and the Revised ISS (R-ISS) (17). Cytogenetic risk was assessed using fluorescence *in situ* hybridization (FISH) for high-risk abnormalities, including del(13q14), del(1p32), gain(1q21), del(17p), Rb deletion, and IgH translocations. These findings were then taken into consideration for the determination of the R-ISS stage (18, 19).

Treatment regimens were also collected and categorized. Induction therapy included the use of proteasome inhibitors (PIs), immunomodulatory drugs (IMiDs), daratumumab (Dara), and cyclophosphamide (CTX). The intensity of induction therapy was defined as the number of drugs used in the regimen: two−drug induction therapy regimen (BD: bortezomib + dexamethasone), three−drug induction therapy regimen (VRD/VPD/VCD/KRD/DVD/DKD), or four−drug induction therapy regimen (KRCD/DVRD/DKPD). Subsequent therapy included autologous stem cell transplantation (ASCT) and maintenance therapy. Maintenance therapy was classified as single−agent (lenalidomide/pomalidomide/dexamethasone, R/P/D) or two−agent (VR/KR/D−V/D−R) regimens.

Treatment response was evaluated according to the IMWG 2016 criteria (17). Minimal residual disease (MRD) was assessed by next-generation flow cytometry (NGF) with a sensitivity threshold of 10^-5^ (20). Progression-free survival (PFS) was defined as the time from treatment initiation to documented disease progression or death from any cause. Overall survival (OS) was defined as the time from treatment initiation to death from any cause. Those without an event were censored at the last follow-up (July 31, 2025).

### 2.3 FCRL5 detection and patient grouping

Pretreatment bone marrow samples were stained with a pre-mixed antibody panel against CD45 (Tonbo Biosciences Cat# 65-0459, RRID: AB_2621897), CD38 (BioLegend Cat# 356626, RRID: AB_2616713), CD138 (BioLegend Cat# 352308, RRID: AB_10896946), and FCRL5 (BD Biosciences Cat# 749608, RRID: AB_2873910). Data acquisition was performed on a BD LSRFortessa flow cytometer (BD Biosciences, San Jose, CA). Data processing and gating were performed using FlowJo software (TreeStar, Ashland, OR). Then, malignant plasma cells were identified and gated by their characteristic CD45^low^CD38^high^ immunophenotype. The FCRL5 positivity rate was defined as the percentage of plasma cells with FCRL5 fluorescence intensity exceeding that of 99% of isotype control-stained cells. All flow cytometric analyses were performed by a single experienced technician to ensure consistency. Because no validated prognostic cutoff for FCRL5 in NDMM currently exists, we used the median FCRL5 positivity rate (75.15%) within our cohort for exploratory patient stratification. This divided patients into FCRL5 high-expression (≥75.15%) and low-expression (<75.15%) groups.

### 2.4 Statistical analysis

Statistical analyses were conducted with SPSS v.27.0 software (IBM Corp., Armonk, NY, USA) for Windows. Using the Shapiro–Wilk test was the distribution of continuous variables evaluated. Median with interquartile range (IQR) was used to present non-normally distributed variables, while the Mann-Whitney U test was employed for their comparison. Summary of categorical variables was presented as frequencies and percentages (n, %), while their comparison was performed using the Chi-square test or Fisher’s exact test for cases with small-sized samples.

Survival outcomes were estimated using the Kaplan–Meier method, and intergroup differences assessed via the log-rank test. Univariate Cox proportional hazards regression was initially utilized to assess potential prognostic factors. Statistically significant variables (*P* < 0.05) were included in a multivariate Cox model subsequently to pinpoint independent prognostic factors. Hazard ratios (HRs) with their respective 95% confidence intervals (CIs) are presented as the primary results, with statistical significance established at a two-sided *P*-value < 0.05.

## 3 Results

### 3.1 Baseline data

A cohort of 54 patients with NDMM were enrolled in the study. Included in the cohort were 27 male and 27 female patients (50.00% each), with a median age of 65 years (range, 60–75). The most prevalent subtype was IgG (57.41%), while half of the patients (50.00%) presenting with ISS Stage III disease. EMD was present in 10 patients (18.52%). Furthermore, cytogenetic data were available for 38 patients (70.37%), among whom 1q21 gain was detected in 15 cases (39.47%). Interestingly, no significant correlation was observed between FCRL5 expression and established high-risk cytogenetic abnormalities (*P* > 0.05; **Table 1**), suggesting that FCRL5 may represent a distinct biological feature independent of conventional genetic risks.

**Table 1:** Comparison of baseline characteristics between FCRL5 low-expression and high-expression groups in patients with newly diagnosed multiple myeloma (NDMM).

Baseline characteristics	Total (n = 54)	FCRL5 low-expression group (n = 27)	FCRL5 high-expression group (n = 27)	*χ*²/*z* value	*P*-value
FCRL5 positivity rate [%, *M* (*Q1*, *Q3*)]	75.15 (51.73,92.75)	52.90 (33.30,62.70)	92.70 (87.90,96.40)	*Z* = -6.31	<0.001
Sex [n (%)]				*χ*²=0.07	0.785
Female	27 (50.00)	14 (51.85)	13 (48.15)		
male	27 (50.00)	13 (48.15)	14 (51.85)		
Age [n (%)]				*χ*²=0.00	1.000
<65 years	26 (48.15)	13 (48.15)	13 (48.15)		
≥65 years	28 (51.85)	14 (51.85)	14 (51.85)		
Hb [n (%)]				*χ*²=0.32	0.573
<100 g/L	34 (62.96)	16 (59.26)	18 (66.67)		
≥100 g/L	20 (37.04)	11 (40.74)	9 (33.33)		
PLT [n (%)]				*χ*²=1.96	0.161
<100 ×10^9^/L	10 (18.52)	7 (25.93)	3 (11.11)		
≥100 ×10^9^/L	44 (81.48)	20 (74.07)	24 (88.89)		
Alb [n (%)]				*χ*²=0.15	0.702
<30 g/L	8 (14.81)	3 (11.11)	5 (18.52)		
≥30 g/L	46 (85.19)	24 (88.89)	22 (81.48)		
Ca²^+^ [n (%)]				*χ*²=0.49	0.484
<2.65 mmol/L	44 (81.48)	21 (77.78)	23 (85.19)		
≥2.65 mmol/L	10 (18.52)	6 (22.22)	4 (14.81)		
Cr [n (%)]				*χ*²=0.00	1.000
<177 μmol/L	44 (81.48)	22 (81.48)	22 (81.48)		
≥177 μmol/L	10 (18.52)	5 (18.52)	5 (18.52)		
β2-MG [n (%)]				*χ*²=0.67	0.413
<5.5 mg/L	25 (46.30)	14 (51.85)	11 (40.74)		
≥5.5 mg/L	29 (53.70)	13 (48.15)	16 (59.26)		
LDH [n (%)]				*χ*²=0.00	1.000
<250 U/L	47 (87.04)	24 (88.89)	23 (85.19)		
≥250 U/L	7 (12.96)	3 (11.11)	4 (14.81)		
BMPC [n (%)]				*χ*²=0.10	0.750
<50%	41 (75.93)	21 (77.78)	20 (74.07)		
≥50%	13 (24.07)	6 (22.22)	7 (25.93)		
Immunoglobulin subtype [n (%)]				–	1.000
IgG type	31 (57.41)	16 (59.26)	15 (55.56)		
IgA type	15 (27.78)	7 (25.93)	8 (29.63)		
Light chain type	8 (14.81)	4 (14.81)	4 (14.81)		
ISS stage [n (%)]				–	0.874
Stage I	9 (16.67)	4 (14.81)	5 (18.52)		
Stage II	18 (33.33)	10 (37.04)	8 (29.63)		
Stage III	27 (50.00)	13 (48.15)	14 (51.85)		
R-ISS stage [n (%)]				–	0.554
Stage I	8 (14.81)	3 (11.11)	5 (18.52)		
Stage II	36 (66.67)	20 (74.07)	16 (59.26)		
Stage III	10 (18.52)	4 (14.81)	6 (22.22)		
EMD [n (%)]	10 (18.52)	5 (18.52)	5 (18.52)	*χ*²=0.00	1.000
Cytogenetic abnormalities [n (%)] ^a^					
Del (13q14)	15 (39.47)	10 (47.62)	5 (29.41)	–	0.326
Del (1p32)	4 (10.53)	1 (4.76)	3 (17.65)	–	0.307
Gain (1q21)	15 (39.47)	7 (33.33)	8 (47.06)	–	0.509
Del (17p)	6 (15.79)	3 (14.29)	3 (17.65)	–	1.000
Rb deletion	16 (42.11)	10 (47.62)	6 (35.29)	–	0.521
IgH rearrangement	29 (76.32)	16 (76.19)	13 (76.47)	–	1.000

Hb, Hemoglobin; PLT, platelets; Alb, Albumin; Ca²^+^, serum calcium; Cr, creatinine; β2-MG, β2-microglobulin; LDH, lactate dehydrogenase; BMPC, bone marrow plasma cell proportion; ISS, International Staging System; R-ISS, Revised International Staging System; EMD, extramedullary disease.

Data are presented as n (%) or Median (*Q1*, *Q3*). Continuous variables were compared using the Mann-Whitney U test, and categorical variables were compared using the Chi-square test or Fisher’s exact test, as appropriate. A dash (“-”) indicates that the *P*-value was calculated using Fisher’s exact test due to an expected frequency <5. *P* < 0.05 was considered statistically significant.

^a^Cytogenetic results were evaluable in 38 patients.

The median FCRL5 positivity among bone marrow plasma cells in the 54 patients was 75.15% (IQR, 51.73 - 92.75). Using this median as the cutoff, patients were stratified into the FCRL5 low-expression (n = 27) and high-expression (n = 27) groups, with a confirmed significant difference in FCRL5 expression levels between the groups (*P* < 0.001). Baseline characteristics were comparable between the two groups across sex, age, key laboratory parameters, disease stage, and cytogenetic risk profiles (all *P* > 0.05). Although serum β2-MG levels were numerically higher in the FCRL5 high-expression patients, suggesting a possible trend toward increased tumor burden, but this difference failed to reach statistical significance (**Table 1**).

Treatment regimens received by the two groups were also compared. As presented in **Table 2**, there were no significant differences between the FCRL5 low−expression and high−expression groups regarding the use of specific induction agents (proteasome inhibitors, immunomodulatory drugs, daratumumab, or cyclophosphamide), the intensity of induction therapy (two−, three−, or four−drug combinations), subsequent autologous stem cell transplantation (ASCT), or maintenance therapy (single−agent or two−agent) (all *P* > 0.05). The distribution of treatment-related variables did not differ statistically between the two groups, although imbalance could not be completely excluded because of the small sample size.

**Table 2:** Comparison of treatment regimens between FCRL5 low-expression and high-expression groups in NDMM patients.

Treatment modality	Total (n = 54)	FCRL5 low-expression group (n = 27)	FCRL5 high-expression group (n = 27)	*χ*² value	*P*-value
Specific agents used in induction therapy [n (%)]					
PIs	54 (100.00)	27 (100.00)	27 (100.00)	–	1.000
IMiDs	44 (81.48)	20 (74.07)	24 (88.89)	*χ*²=1.96	0.161
Dara	11 (20.37)	4 (14.81)	7 (25.93)	*χ*²=1.03	0.311
CTX	5 (9.26)	3 (11.11)	2 (7.41)	*χ*²=0.00	1.000
Intensity of induction therapy [n (%)]				–	0.149
Two−drug induction therapy regimen (BD) [n (%)]	2 (3.70)	2 (7.41)	0 (0.00)		
Three-drug induction therapy regimen (VRD/VPD/VCD/KRD/DVD/DKD) [n (%)]	44 (81.48)	23 (85.19)	21 (77.78)		
Four-drug induction therapy regimen (KRCD/DVRD/DKPD) [n (%)]	8 (14.81)	2 (7.41)	6 (22.22)		
Subsequent therapy [n (%)]					
ASCT	17 (31.48)	9 (33.33)	8 (29.63)	*χ*²=0.09	0.770
No maintenance therapy	5 (9.26)	3 (11.11)	2 (7.41)	*χ*²=0.00	1.000
Single−agent maintenance (R/P/D)	27 (50.00)	15 (55.56)	12 (44.44)	*χ*²=0.67	0.414
Two−agent maintenance (VR/KR/D−V/D−R)	22 (40.74)	9 (33.33)	13 (48.15)	*χ*²=1.23	0.268

NDMM, newly diagnosed multiple myeloma; PIs, proteasome inhibitors; IMiDs, immunomodulatory drugs; Dara, daratumumab; CTX, cyclophosphamide; ASCT, autologous stem cell transplantation.

BD, bortezomib + dexamethasone; VRD, bortezomib + lenalidomide + dexamethasone; VPD, bortezomib + pomalidomide + dexamethasone; VCD, bortezomib + cyclophosphamide + dexamethasone; KRD, carfilzomib + lenalidomide + dexamethasone; DVD, daratumumab + bortezomib + dexamethasone; DKD, daratumumab + carfilzomib + dexamethasone; KRCD, carfilzomib + lenalidomide + cyclophosphamide + dexamethasone; DVRD, daratumumab + bortezomib + lenalidomide + dexamethasone; DKPD, daratumumab + carfilzomib + pomalidomide + dexamethasone; R, lenalidomide; P, pomalidomide; D, dexamethasone; VR, bortezomib + lenalidomide; KR, carfilzomib + lenalidomide; D−V, daratumumab + bortezomib; D−R, daratumumab + lenalidomide.

Between-group comparisons were performed using the Chi-square or Fisher’s exact test, as appropriate. A dash (“-”) denotes results derived from Fisher’s exact test due to expected frequencies <5. *P* < 0.05 was considered statistically significant.

### 3.2 Treatment response

All enrolled patients completed four cycles of induction therapy based on immunomodulatory agents and/or proteasome inhibitors. There was no statistically significant difference in response rates between the two groups after two cycles (*P* = 0.669). However, after four cycles, FCRL5 high-expression patients had a significant worse response (*P* = 0.046). Specifically, complete response (CR; 25.93% vs 51.85%, *P* = 0.046), very good partial response or better (≥VGPR; 33.33% vs 70.37%, *P* = 0.006), and MRD negativity (29.63% vs 70.37%, *P* = 0.003) were significantly less frequent in the FCRL5 high-expression group than in the low-expression group. Despite a high overall response rate (ORR) in both groups, the two cohorts were comparable in terms of ORR (100% vs 88.89%, *P* = 0.235). Collectively, these observations demonstrate a tentative association between low FCRL5 expression and deeper treatment responses after four cycles of induction therapy in the present study (**Table 3**).

**Table 3:** Comparison of treatment response after two and four cycles of induction therapy between FCRL5 low-expression and high-expression groups.

Response evaluation	Total (n = 54)	FCRL5 low-expression group (n = 27)	FCRL5 high-expression group (n = 27)	*χ*² value	*P*-value
Response after two cycles [n (%)]				–	0.669
CR	10 (18.52)	6 (22.22)	4 (14.81)		
VGPR	3 (5.56)	2 (7.41)	1 (3.70)		
PR	32 (59.26)	16 (59.26)	16 (59.26)		
SD	9 (16.67)	3 (11.11)	6 (22.22)		
Response after four cycles [n (%)]				–	0.046
CR	21 (38.89)	14 (51.85)	7 (25.93)		
VGPR	7 (12.96)	5 (18.52)	2 (7.41)		
PR	23 (42.59)	8 (29.63)	15 (55.56)		
SD	1 (1.85)	0 (0.00)	1 (3.70)		
PD	2 (3.70)	0 (0.00)	2 (7.41)		
ORR [n (%)]	51 (94.44)	27 (100.00)	24 (88.89)	*χ*²=1.41	0.235
≥VGPR [n (%)]	28 (51.85)	19 (70.37)	9 (33.33)	*χ*²=6.98	0.006
MRD negativity [n (%)]	27 (50.00)	19 (70.37)	8 (29.63)	*χ*²=8.04	0.003

CR, complete response; VGPR, very good partial response; PR, partial response; SD, stable disease; PD, progressive disease; ORR, overall response rate (CR+VGPR+PR); ≥VGPR, very good partial response or better; MRD, minimal residual disease.

Between-group comparisons were performed using the Chi-square or Fisher’s exact test, as appropriate. A dash (“-”) denotes results derived from Fisher’s exact test due to expected frequencies <5. *P* < 0.05 was considered statistically significant.

Among the 27 patients in the high-expression group, treatment regimens were further categorized into two subgroups: the three-drug combination therapy group (n = 21) and the four-drug combination therapy group (n = 6). The efficacy of the two regimens was compared within this subgroup. After six months of follow-up, treatment with the four-drug regimen was associated with higher rates of ORR (100.00% vs 80.95%), ≥VGPR (83.33% vs 33.33%), CR (50.00% vs 19.05%), and MRD negativity (100.00% vs 61.90%) compared to the three-drug regimen. Two patients in the three-drug therapy group experienced disease progression, with no progression occurred in the four-drug therapy group. It revealed a tentative trend toward improved outcomes with the four-drug regimen relative to the three-drug regimen in this cohort (**Table 4**).

**Table 4:** Treatment response to different multi-drug induction regimens in NDMM patients with high FCRL5 expression.

Response evaluation [n (%)]	Total (n=27)	Three-drug (n=21)	Four-drug (n=6)
CR	7 (25.93)	4 (19.05)	3 (50.00)
VGPR	5 (18.52)	3 (14.29)	2 (33.33)
PR	11 (40.74)	10 (47.62)	1 (16.67)
SD	2 (7.41)	2 (9.52)	0 (0.00)
PD	2 (7.41)	2 (9.52)	0 (0.00)
ORR	23 (85.19)	17 (80.95)	6 (100.00)
≥VGPR	12 (44.44)	7 (33.33)	5 (83.33)
MRD negativity	19 (70.37)	13 (61.90)	6 (100.00)

CR, complete response; VGPR, very good partial response; PR, partial response; SD, stable disease; PD, progressive disease; ORR, overall response rate (CR+VGPR+PR); ≥VGPR, very good partial response or better; MRD, minimal residual disease.

Data are presented as n (%). No statistical analysis was performed due to the limited sample size in this subgroup.

### 3.3 Survival outcomes

**Figure 1:**
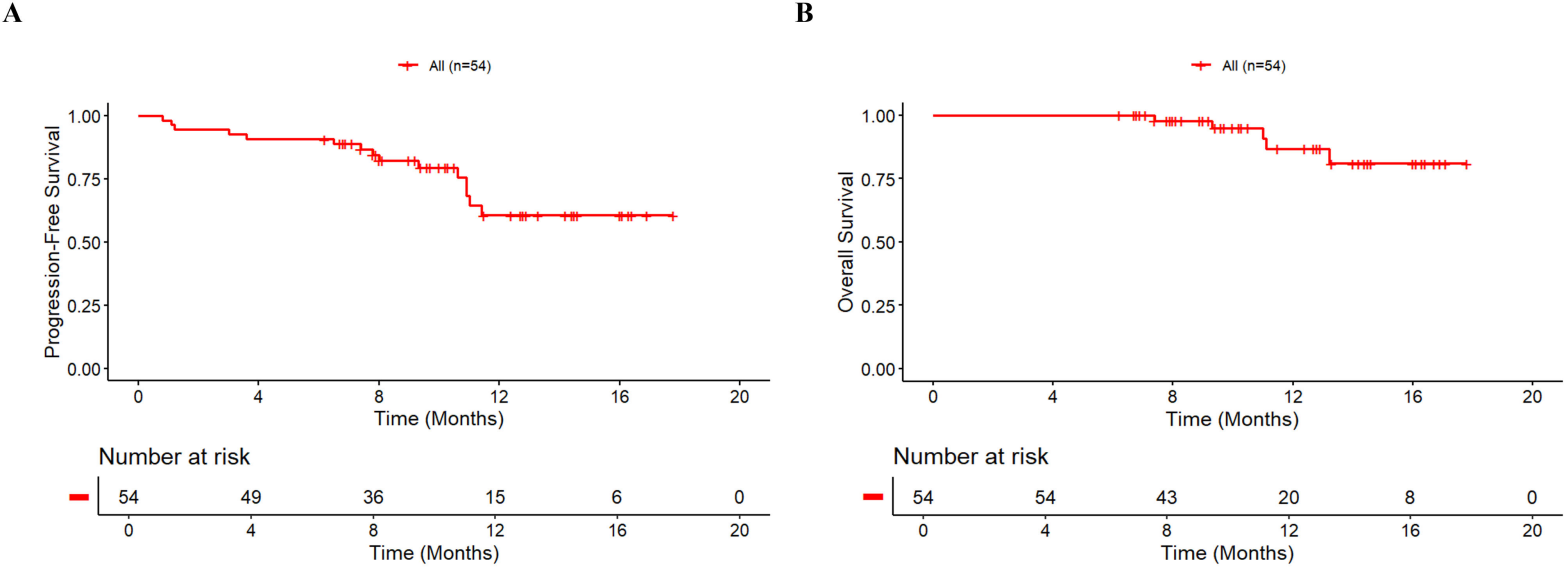
Kaplan-Meier survival analysis for the entire cohort (n=54). **(A)** Kaplan-Meier curve of progression-free survival (PFS); **(B)** Kaplan-Meier curve of overall survival (OS).

**Figure 2:**
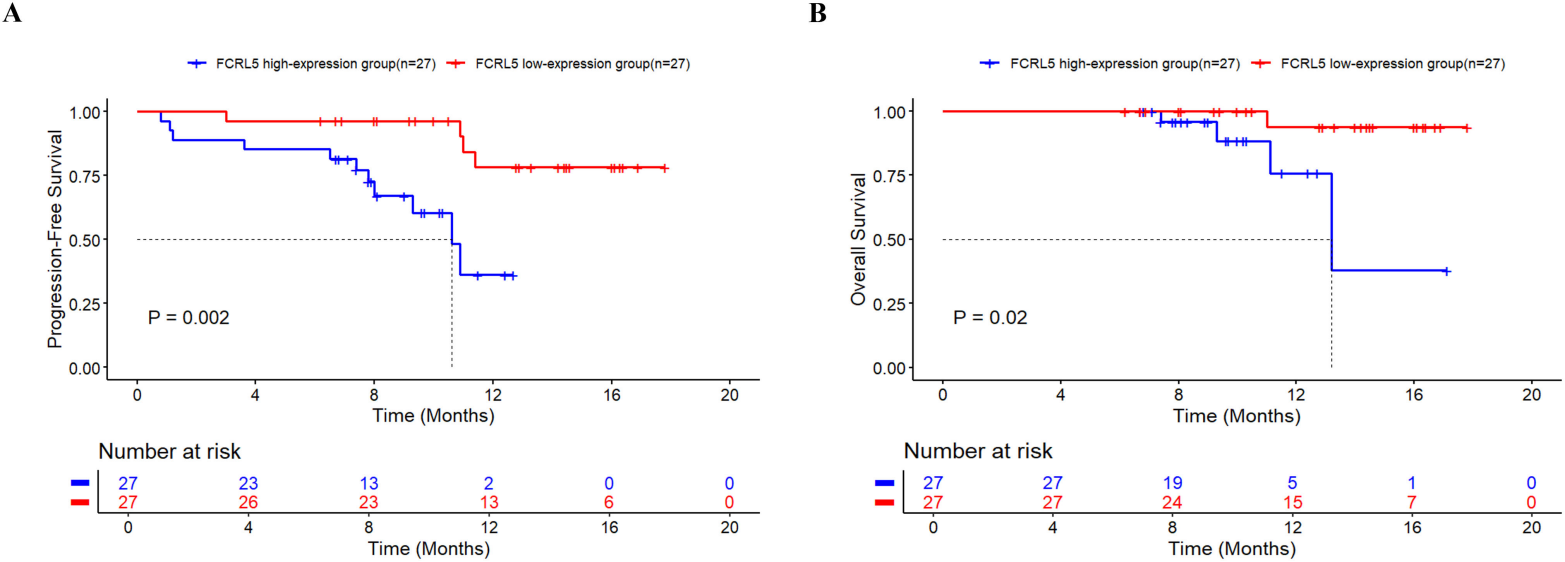
Impact of FCRL5 expression on survival outcomes in patients with newly diagnosed multiple myeloma. **(A)** Kaplan-Meier curve of progression-free survival (PFS) in the FCRL5 high-expression (≥75.15%) and low-expression (<75.15%) groups; **(B)** Kaplan-Meier curve of overall survival (OS) in the FCRL5 high-expression (≥75.15%) and low-expression (<75.15%) groups.

To further investigate the potential correlation between FCRL5 expression and survival outcomes in NDMM patients, a survival analysis was performed on the 54 patients. As of July 31, 2025, the median follow-up was 10.3 months (95% *CI*: 9.506 - 11.094) for all patients. The survival analysis revealed that median PFS and OS remained unreached in the entire study cohort. One-year PFS and OS rates were 60.6% and 86.8%, respectively (**Figure 1**). Median PFS was significantly shorter in the FCRL5 high-expression group than in the low-expression group (10.6 months vs. not reached, *P* = 0.002). A similar trend was noted for median OS, with shorter median OS in the FCRL5 high-expression group (13.2 months vs. not reached, *P* = 0.020). Regarding survival probabilities, the FCRL5 high-expression group had a significantly lower one-year PFS rate (21.43% vs. 76.47%, *P* = 0.004), whereas no statistically significant difference was observed in the one-year OS rate between the two groups (66.67% vs. 93.75%, *P* = 0.116) (**Figure 2**).

### 3.4 Prognostic factor analysis

Univariate Cox regression was utilized to further assess factors associated with PFS and OS in 54 NDMM patients, based on their baseline characteristics and laboratory parameters. The univariate analysis revealed that high FCRL5 expression, elevated Cr, elevated β2-MG, and the presence of EMD each exhibited a statistically significant association with shorter PFS in NDMM patients (all *P* < 0.05), suggesting these factors may influence disease progression. In contrast, induction therapy agents, ASCT, and maintenance therapies were not significantly associated with PFS in the univariate analysis (all *P* > 0.05). These factors that reached statistical significance in the univariate analysis were then included in a multivariate Cox regression model, which identified high FCRL5 expression (*P* = 0.004), elevated β2-MG (*P* = 0.018), and the presence of EMD (*P* = 0.035) as independent predictors of shorter PFS in this study cohort (**Table 5**).

**Table 5:** Univariate and multivariate Cox regression analyses of factors associated with progression-free survival (PFS) in NDMM patients.

Clinical characteristic	Univariate analysis			Multivariate analysis		
	*HR*	95%*CI*	*P*-value	*HR*	95%*CI*	*P*-value
FCRL5 high expression	5.52	1.68~18.12	0.005	7.32	1.86~28.74	0.004
Sex (Male)	1.20	0.43~3.31	0.729	–	–	–
Hb (<100 g/L)	1.70	0.54~5.36	0.363	–	–	–
PLT (<100 ×10^9^/L)	1.13	0.36~3.58	0.832	–	–	–
Alb (<30 g/L)	1.49	0.42~5.30	0.535	–	–	–
Ca²^+^ (≥2.65 mmol/L)	2.37	0.81~6.97	0.116	–	–	–
Cr (≥177 μmol/L)	3.41	1.10~10.54	0.033	0.74	0.18~3.09	0.676
β2-MG (≥5.5 mg/L)	4.76	1.33~17.06	0.017	5.47	1.33~22.43	0.018
LDH (≥250 U/L)	3.06	0.97~9.63	0.056	–	–	–
BMPC (≥50%)	1.69	0.58~4.96	0.336	–	–	–
EMD	2.85	1.01~8.04	0.048	3.79	1.10~13.09	0.035
Four-drug induction therapy regimen	0.43	0.06~3.28	0.416			
IMiDs (induction therapy)	0.65	0.21~2.05	0.463			
Dara (induction therapy)	0.72	0.16~3.17	0.660			
CTX (induction therapy)	1.07	0.24~4.79	0.927			
ASCT	0.97	0.33~2.83	0.951			
Single−agent maintenance	0.96	0.35~2.66	0.934			
Two−agent maintenance	0.52	0.16~1.63	0.262			

HR, hazard ratio; *CI*, confidence interval; Hb, Hemoglobin; PLT, platelets; Alb, Albumin; Ca²^+^, serum calcium; Cr, creatinine; β2-MG, β2-microglobulin; LDH, lactate dehydrogenase; BMPC, bone marrow plasma cell proportion; EMD, extramedullary disease; IMiDs, immunomodulatory drugs; Dara, daratumumab; CTX, cyclophosphamide; ASCT, autologous stem cell transplantation.

IMiDs, Dara, and CTX were used as induction therapy. ASCT was administered as subsequent therapy, and single-agent or two-agent maintenance was given as maintenance therapy.

Variables with a *P*-value < 0.05 in the univariate analysis were included in the multivariate model. A dash (“-”) indicates variables excluded from the multivariate analysis. *P* < 0.05 was considered statistically significant. *HR* > 1 indicates increased risk.

Univariate Cox regression for OS, performed using the same methodology, demonstrated that both high FCRL5 expression and elevated Cr were significantly associated with shorter OS in NDMM patients (both *P* < 0.05). Other variables, induction therapy agents, ASCT, and maintenance therapies, showed no significant association with OS in the univariate analysis (all *P* > 0.05). Confirmed by further multivariate Cox regression analysis high FCRL5 expression (*P* = 0.049) was an independent adverse prognostic factor for OS in this single-center cohort (**Table 6**). Together, these exploratory analyses in our small, single-center cohort suggest that high FCRL5 expression may be an independent prognostic factor for both disease progression and mortality in NDMM.

**Table 6:** Univariate and multivariate Cox regression analyses of factors associated with overall survival (OS) in NDMM patients.

Clinical characteristic	Univariate analysis			Multivariate analysis		
	*HR*	95%*CI*	*P*-value	*HR*	95%*CI*	*P*-value
FCRL5 high expression	9.82	1.01~95.10	0.049	9.82	1.01~95.10	0.049
Sex (Male)	4.32	0.48~38.77	0.192	–	–	–
Age (≥65 years)	1.82	0.30~10.93	0.512	–	–	–
PLT (<100 ×10^9^/L)	1.81	0.30~10.94	0.519	–	–	–
Ca²^+^ (≥2.65 mmol/L)	1.00	0.11~8.98	0.999	–	–	–
Cr (≥177 μmol/L)	7.90	1.30~48.06	0.025	–	–	–
LDH (≥250 U/L)	1.46	0.16~13.18	0.735	–	–	–
BMPC (≥50%)	2.03	0.34~12.20	0.440	–	–	–
IMiDs (induction therapy)	0.58	0.09~3.60	0.560			
Dara (induction therapy)	0.93	0.10~8.33	0.949			
CTX (induction therapy)	1.36	0.15~12.37	0.783			
ASCT	0.46	0.05~4.15	0.492			
Single−agent maintenance	0.64	0.11~3.86	0.631			
Two−agent maintenance	0.41	0.05~3.70	0.429			

*HR*, hazard ratio; *CI*, confidence interval; PLT, platelets; Ca²^+^, serum calcium; Cr, creatinine; LDH, lactate dehydrogenase; BMPC, bone marrow plasma cell proportion; IMiDs, immunomodulatory drugs; Dara, daratumumab; CTX, cyclophosphamide; ASCT, autologous stem cell transplantation.

IMiDs, Dara, and CTX were used as induction therapy. ASCT was administered as subsequent therapy, and single-agent or two-agent maintenance was given as maintenance therapy.

Variables with a *P*-value < 0.05 in the univariate analysis were included in the multivariate model. A dash (“-”) indicates variables excluded from the multivariate analysis. *P* < 0.05 was considered statistically significant. *HR* > 1 indicates increased risk.

## 4 Discussion

MM remains incurable because of its considerable heterogeneity, which manifests from the initial clinical presentation through to long-term outcomes. This variability highlights the pressing demand for accurate prognostic instruments to facilitate personalized therapeutic regimens in clinical practice (21). Currently, contemporary risk assessment primarily relies on traditional cytogenetics and a few surface markers such as BCMA and GPRC5D. While significantly improving patient management, its predictive capabilities remain limited and may fail to fully reflect the biological complexity and individual variability of the disease (22, 23). In response to this critical gap, the identification of novel biomarkers with independent prognostic value has become a research priority, with FCRL5 emerging as a potential candidate for further evaluation.

Exhibiting minimal expression in normal hematopoietic tissues, FCRL5 is a transmembrane signaling protein that demonstrates highly specific expression on myeloma cells (10). Such markedly differential expression of FCRL5 supports its potential for tumor-selective targeting in MM immunotherapy, which is anticipated to minimize on-target, off-tumor toxicity (6). Beyond its established role in humoral immunity (12), FCRL5 can also act as an IgG receptor, exhibiting substantially enhanced binding affinity in MM (24). As a key transmembrane signaling protein in plasma cell development, FCRL5 may also mediate the clonal evolution of plasma cells in the early stage of myeloma pathogenesis (25). This process may be related to the role of FCRL5 in promoting plasma cell survival and resisting the innate immune surveillance of the bone marrow microenvironment, which provides a potential mechanism for the early development of drug resistance and disease progression in MM. However, the specific regulatory network needs to be verified by subsequent longitudinal cohort studies and functional assays (9, 13). Consequently, several therapeutic strategies targeting FCRL5, such as ADCs, bispecific antibodies, and CAR-T therapies, are currently undergoing active preclinical research and have shown promising activity against myeloma (14, 26, 27). However, their clinical utility remains to be fully established by further systematic validation and real-world evidence.

In this retrospective study, we systematically compared clinical characteristics, treatment regimens, treatment response, and survival outcomes across NDMM patients stratified by FCRL5 expression levels to clarify the clinical value of FCRL5. Regarding clinical features, our study identified that FCRL5 may display an independent predictive role in MM, predominantly driven by its selective expression and functional characteristics. Aligning with early reports linking FCRL5 expression to B-cell developmental stage rather than demographic factors or disease stage (8, 28), we observed that its overexpression was not directly associated with traditional adverse prognostic factors such as elevated Cr, β2-MG, or EMD (17, 18). This finding suggests that FCRL5 could provide prognostic value complementary to conventional biomarkers, thereby adding a new dimension to risk stratification in NDMM.

Notably, our study found no significant association between FCRL5 expression and established high-risk cytogenetic features such as del17p and 1q21 amplification in NDMM patients. This lack of correlation suggest that FCRL5 may capture an orthogonal dimension of risk – one that is reflected by current cytogenetic panels (29). Given the profound heterogeneity of NDMM, FCRL5 expression might be linked to specific aspects of clonal architecture or the immune contexture within the bone marrow niche that are independent of large-scale genomic alterations. For instance, high FCRL5 expression could characterize aggressive sub-clones that drive early relapse even in patients traditionally classified as standard-risk. While previous reports suggest epigenetic mechanisms might regulate FCRL5, our findings emphasize that FCRL5 reflects the biological complexity of myeloma from a perspective distinct from traditional cytogenetics (14, 30). From a clinical standpoint, this observation raises the possibility that FCRL5 expression might have a role in risk refinement, but whether it helps identify patients with suboptimal outcomes in the absence of high−risk cytogenetic lesions remains to be determined. In addition, the correlation between FCRL5 expression and the immune contexture of the bone marrow microenvironment, such as the proportion of Treg cells, M2-type macrophages, remains to be explored, which is an important direction to further clarify the role of FCRL5 in myeloma heterogeneity. However, this hypothesis is exploratory and requires further validation through single-cell sequencing or integrated immune profiling to clarify how FCRL5-high cells interact with the tumor microenvironment.

Building on the independent prognostic association identified above, we further explored whether high FCRL5 expression acts as a favorable or unfavorable prognostic factor in MM. Similar to findings described in certain solid tumors to hematological malignancies (31–33), we speculate from our survival analysis that high FCRL5 expression may act as a potential predictor of adverse prognosis in MM, associated with reduced PFS and OS. However, given the relatively small sample size and short follow-up duration, the observed differences in OS outcomes should be interpreted with caution, as the median OS has not yet been reached in the low-expression group. This finding gains additional support from emerging therapeutic insights. Niu et al. (13) reported marked FCRL5 upregulation in BCMA-targeted CAR-T relapsed MM, correlating with faster progression and poorer survival, confirming its dual role as a prognostic indicator and potential target. However, these findings are limited by our small sample size and thus require validation in multicenter, large-sample prospective clinical studies.

At present, standard first-line induction therapy for NDMM typically adopts triple-drug combination based on immunomodulatory agents and proteasome inhibitors. Sometimes, clinicians will also choose to use a quadruple-drug combination with monoclonal antibody. These drugs mainly exert anti-tumor effects through three key mechanisms: enhanced immune surveillance against tumor cells, induction of proteotoxic stress within malignant plasma cells, and coordinated activation of apoptotic pathways (34, 35). Although treatment-related variables were not statistically different between the two FCRL5 groups, residual confounding due to treatment heterogeneity cannot be excluded. Nevertheless, FCRL5-high patients showed inferior response depth and survival in this exploratory cohort. Prior research suggests that elevated FCRL5 may reduce plasma cell chemosensitivity, potentially explaining inferior treatment response in FCRL5-high patients (36). Accordingly, our data suggest that FCRL5 expression may be correlated with treatment resistance, although the underlying biological mechanisms remain to be fully elucidated via functional experiments. These findings highlight the potential for developing risk-adapted strategies, where early identification of FCRL5-high patients might provide a rationale for exploring more intensive induction approaches in future studies.

Through further subgroup analysis of patients with high expression of FCRL5, we noted that the ORR and 6-month MRD negative rate reached 100% in the small subset of patients receiving the quadruplet regimen (n=6). But this finding is purely exploratory due to the extremely small sample size and inherent treatment heterogeneity in the current cohort, and no firm conclusions about the comparative efficacy of the quadruplet versus triple-drug regimen can be drawn from these limited data. Furthermore, we preliminarily observed a tentative trend that patients receiving longer induction may show better response and remission depth, which warrants further investigation. If validated in larger studies, this trend may be particularly relevant for transplant-eligible FCRL5-high patients, as deep cytoreduction and MRD negativity before autologous stem cell transplantation (ASCT) are key clinical goals linked to improved post-transplant outcomes and long-term survival in multiple myeloma.

Due to the treatment heterogeneity and the small number of patients in specific subgroups, no firm conclusions can be drawn regarding the definitive superiority of quadruplet regimens over triple-drug combinations based on the current cohort. Therefore, while preliminary, our findings support a tentative hypothesis that more intensive induction strategies, such as quadruplet regimens and extended induction courses, could potentially confer clinical benefits for FCRL5-high NDMM patients. For those with suboptimal responses to such intensified induction, future investigations could explore whether earlier integration of novel immunotherapies may improve clinical outcomes in this subgroup. Given the exploratory nature of our data, these findings should be regarded as hypothesis-generating rather than definitive. The clinical utility of FCRL5 for risk-adapted therapy requires rigorous validation in prospective, multicenter studies with standardized assays and longer follow-up periods.

Several limitations should be acknowledged in the present study. Firstly, the small sample size and single-center setting may limit the generalizability of our findings. Secondly, the median FCRL5 positivity rate (75.15%) employed for patient stratification is exploratory in nature. To date, there is no validated clinical cutoff value established for FCRL5 in MM. The optimal clinical cutoff value for FCRL5 requires further determination through large-sample, multi-center validation studies employing standardized flow cytometry assays. Thirdly, due to the lack of functional and correlative immune analyses, the potential biological mechanism of FCRL5 in MM progression and treatment resistance can only be regarded as a hypothesis, and no causal inference can be made. Fourthly, the lack of integrated analysis of FCRL5 with the bone marrow immune microenvironment and clonal architecture limits the in-depth understanding of its role in myeloma heterogeneity. Moreover, treatment subgroup statistical analysis was omitted due to small cohort sizes that would have compromised statistical power and result persuasiveness. Lastly, the follow-up duration was relatively short coupled with a small sample size, which restricts the reliable interpretation of OS outcomes and limits our ability to comprehensively assess long-term treatment efficacy in general. Subsequent validation therefore necessitates large-scale, multicenter studies with extended follow-up and the sample size.

## 5 Conclusions

In conclusion, our study preliminarily suggests elevated FCRL5 expression is associated with a poor treatment response in NDMM patients. However, given the exploratory nature of our data, these findings should be regarded as hypothesis-generating rather than definitive. While FCRL5 represents a potential biomarker for risk stratification and a promising therapeutic target, its clinical readiness remains to be established. Future research should focus on large-scale, prospective validation with standardized assays to confirm whether FCRL5 can reliably guide therapeutic decision-making.

## Data Availability

The datasets generated and analyzed for this study are not publicly available due to patient privacy and institutional regulations but are available from the corresponding author upon reasonable request. Requests to access these datasets should be directed to liaoyuanxing11@163.com.
